# Heme-Induced Oxidation of Cysteine Groups of Myofilament Proteins Leads to Contractile Dysfunction of Permeabilized Human Skeletal Muscle Fibres

**DOI:** 10.3390/ijms21218172

**Published:** 2020-10-31

**Authors:** Gerardo Alvarado, Attila Tóth, Éva Csősz, Gergő Kalló, Katalin Dankó, Zoltán Csernátony, Ann Smith, Magnus Gram, Bo Akerström, István Édes, György Balla, Zoltán Papp, József Balla

**Affiliations:** 1 HAS-UD Vascular Biology and Myocardial Pathophysiology Research Group, Hungarian Academy of Sciences, H-4032 Debrecen, Hungary; dr.gerardoac@gmail.com (G.A.); atitoth@med.unideb.hu (A.T.); 2 Division of Clinical Physiology, Department of Cardiology, Faculty of Medicine, University of Debrecen, H-4032 Debrecen, Hungary; edes@med.unideb.hu; 3 Proteomics Core Facility, Department of Biochemistry and Molecular Biology, Faculty of Medicine, University of Debrecen, H-4032 Debrecen, Hungary; cseva@med.unideb.hu (É.C.); kallogergo@unideb.hu (G.K.); 4 Department of Rheumatology, Institute of Internal Medicine, Faculty of Medicine, University of Debrecen, H-4032 Debrecen, Hungary; danko.katalin@med.unideb.hu; 5 Department of Orthopedics, Faculty of Medicine, University of Debrecen, H-4012 Debrecen, Hungary; csz@med.unideb.hu; 6 Department of Cell and Molecular Biology and Biochemistry, School of Biological and Chemical Sciences, University of Missouri-Kansas City, Missouri, MO 64110, USA; smithan@umkc.edu; 7 Department of Clinical Sciences Lund, Pediatrics, Lund University, 22184 Lund, Sweden; magnus.gram@med.lu.se; 8 Department of Clinical Sciences Lund, Infection Medicine, Lund University, 22184 Lund, Sweden; bo.akerstrom@med.lu.se; 9 Institute of Pediatrics, Faculty of Medicine, University of Debrecen, H-4012 Debrecen, Hungary; balla@med.unideb.hu; 10 Department of Nephrology, Institute of Medicine, Faculty of Medicine, University of Debrecen, H-4012 Debrecen, Hungary

**Keywords:** skeletal muscle fibre, contractile dysfunction, heme, sulfhydryl groups, sulfenic acid formation, chronic heart failure, oxidation, hemopexin, α1-microglobulin, skeletal muscle myopathy

## Abstract

Heme released from red blood cells targets a number of cell components including the cytoskeleton. The purpose of the present study was to determine the impact of free heme (20–300 µM) on human skeletal muscle fibres made available during orthopedic surgery. Isometric force production and oxidative protein modifications were monitored in permeabilized skeletal muscle fibre segments. A single heme exposure (20 µM) to muscle fibres decreased Ca^2+^-activated maximal (active) force (F_o_) by about 50% and evoked an approximately 3-fold increase in Ca^2+^-independent (passive) force (F_passive_). Oxidation of sulfhydryl (SH) groups was detected in structural proteins (e.g., nebulin, α-actinin, meromyosin 2) and in contractile proteins (e.g., myosin heavy chain and myosin-binding protein C) as well as in titin in the presence of 300 µM heme. This SH oxidation was not reversed by dithiothreitol (50 mM). Sulfenic acid (SOH) formation was also detected in the structural proteins (nebulin, α-actinin, meromyosin). Heme effects on SH oxidation and SOH formation were prevented by hemopexin (Hpx) and α1-microglobulin (A1M). These data suggest that free heme has a significant impact on human skeletal muscle fibres, whereby oxidative alterations in structural and contractile proteins limit contractile function. This may explain and or contribute to the weakness and increase of skeletal muscle stiffness in chronic heart failure, rhabdomyolysis, and other hemolytic diseases. Therefore, therapeutic use of Hpx and A1M supplementation might be effective in preventing heme-induced skeletal muscle alterations.

## 1. Introduction

Skeletal and cardiac muscles share several similarities with regards to their ultrastructure (e.g., cross-striation) and function (e.g., Ca^2+^-regulated contraction). Nevertheless, there are important differences in the molecular composition of their sarcomeric proteins [1]. Myosin heavy chain (MHC) isoforms have a dominant role in the kinetics of muscle contraction in both skeletal and cardiac muscles. Myosin β isoform (β-MHC) expression produces contributes to renders a relatively slow muscle contraction, whereas α-MHC expression leads to fast kinetics to muscle contraction [2]. Additional mechanical differences are/generally considered to be related to titin isoform expressions of skeletal and cardiac muscles [3]. While the role of MHC is central in Ca^2+^ dependent (hence active) force generation [4], titin is vital for the passive tension in striated muscles [5].

Besides titin and MHC, there are numerous additional sarcomeric proteins, such as structural proteins (e.g., nebulin, α-actinin, meromyosin), contractile proteins (e.g., myosin light chains, actin) and regulatory proteins (e.g., troponins) that are essential for the contractile process in striated muscles [6]. It is important to note, that all of these proteins contain sulfhydryl (SH) groups, which can react with different oxidizing agents [7,8]; furthermore, their oxidation may limit contractile performance through a reduction of active force and/or increase in passive tension generation [9].

Heme is present in a number of hemoproteins with essential biological functions [10]. It is involved in energy production (cytochromes) and oxygen transport (myoglobin) in skeletal muscles. Of note, excess free heme evokes DNA damage, lipid peroxidation, mitochondrial dysfunction and protein alterations [11,12,13]. Cellular protective systems prevent the deleterious effects of heme release. Extracellularly, in particular, hemopexin (Hpx) binds to heme with high affinity and removes it from the blood circulation. Elevated heme levels and heme-containing molecules actively stimulate the activity and production of Hpx, as seen in chronic neuromuscular diseases [14]. However, in some disorders such as hemolytic diseases or fulminant rhabdomyolysis, levels of Hpx may become depleted [15]. Additional molecules in biological flus such as plasma that can limit free heme-mediated toxic effects include extracellular plasma high- and low-density lipoproteins (HDL and LDL, respectively) and α1-microglobulin (A1M). Heme toxicity mitigation intracellularly involves heme oxygenase 1 (HO-1) that is involved in heme catabolism [16].

Previous experimental efforts have provided insights into heme induced skeletal muscle alterations [17,18,19]. For example, Lund et al. demonstrated potential cross-links between myosin and activated hemoproteins during in vitro conditions and implicated the involvement of cross-linked myosin molecules in altered muscle function during oxidative stress [17]. In another model study, heme interfered with the polymerization of actin, thereby providing a potential explanation for its toxic effect on rabbit skeletal muscle [18]. Recently, our research group revealed additional molecular links between heme-induced oxidative protein changes and altered cardiac contractile function [19]. Furthermore, heme is implicated in cardiomyopathy in sickle cell disease (SCD) [20] Our current hypothesis stems from the cell and protein similarities between the sarcomeres of cardiac and skeletal muscles. Therefore, we postulate that heme may limit skeletal muscle function through biochemical alterations that are comparable to our previous studies on those presented for cardiac muscles earlier [19].

The present study aimed to determine the effects of free heme on myofibrillar proteins in permeabilized human skeletal muscles fibres. To this end, segments of human skeletal muscle fibres were studied before and after incubations in the presence of heme and two heme scavengers (Hpx and A1M), and deleterious effects on the mechanical function of muscle preparations were monitored. In addition, the level of oxidation (i.e., SH-group oxidation and sulfenylation) of sarcomeric proteins were assayed in parallel biochemical studies, in order to reveal the molecular mechanisms involved in the observed functional alterations. Our results suggest that heme evokes oxidation of cysteine groups of contractile and structural proteins of the sarcomere in skeletal muscles together with sulfenylation of structural muscle proteins causing/contributing to/leading to contractile dysfunction.

## 2. Results

**Figure 1:**
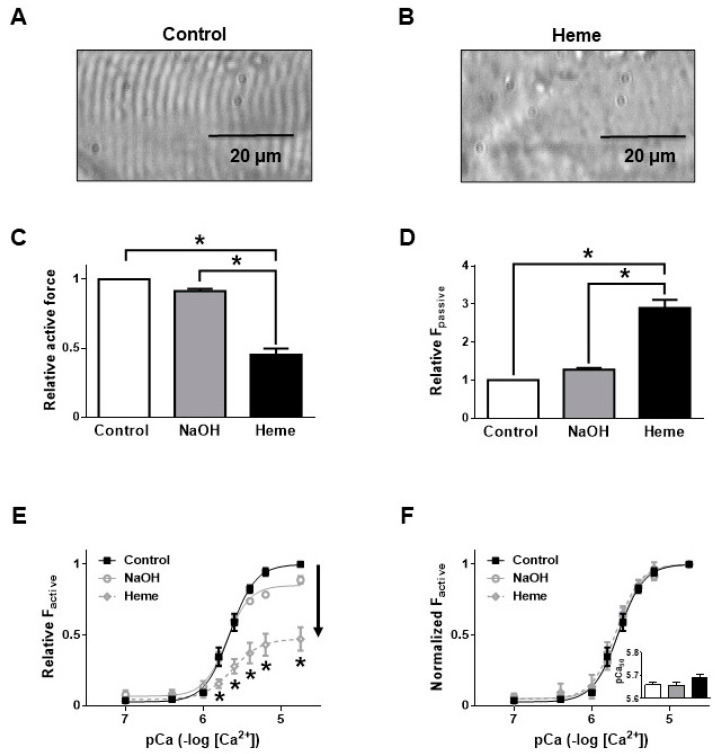
Functional effects of heme on human skeletal muscle fibres. Typical photomicrographs of permeabilized human skeletal muscle fibres. Muscle preparations were incubated in the presence of reaction buffer (**A**) or 20 µM heme (**B**) for 20 min at room temperature (~25 °C). The normal cross striation pattern is clear in the control. Contractile parameters such as Ca^2+^-dependent (active) force (**C**) and Ca^2+^-independent (passive) force (**D**) were determined before and after exposures to the buffer alone, vehicle of heme solvent (NaOH) or heme/NaOH (20 µM). Active force was also plotted as a function of the applied Ca^2+^ concentration. Active force was either normalized to the maximal contractile force before treatments (**E**) or to the maximal contractile force recorded at the highest Ca^2+^ concentration after treatments (**F**). In this latter case the pCa_50_ (i.e., the value representing the Ca^2+^ sensitivity of force production) is also shown in the insert. Bars and symbols show means ± S.E.M. and statistical significance is indicated by asterisks (*) when *p* < 0.05. The number of observations was *n* = 5 for all groups.

Active force at saturating Ca^2+^ concentration (F_o_) was 36.4 ± 3.6 kN/m^2^ in permeabilized skeletal muscle fibre segments before heme exposures. Incubations in the presence of heme (20 µM, 20 min) deteriorated the cross-striation pattern of muscle preparations (Figure 1A,B), decreased F_o_ to 19.8 ± 5.5 kN/m^2^ (45.3 ± 4.4%) (Figure 1C), and increased F_passive_ from 2.7 ± 0.7 kN/m^2^ to 7.4 ± 1.2 kN/m^2^ (269 ± 21.5%) (Figure 1D). The vehicle (NaOH) was without effect (Figure 1C,D). Following heme exposures F_active_ was lower at all investigated Ca^2+^ concentrations (Figure 1E), however, the Ca^2+^ sensitivity of force production (Figure 1F) was unaffected by this concentration of heme.

**Figure 2:**
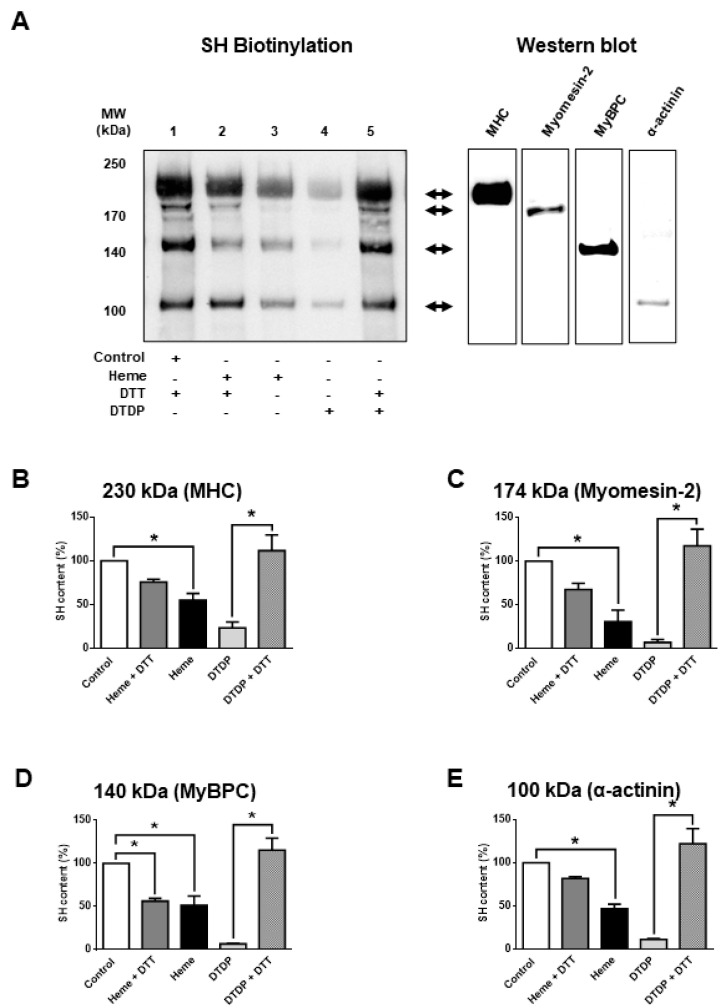
Heme-induced SH-group oxidation. (**A**) Skeletal muscle fibres were isolated and permeabilized as described in the Methods section. About 25 µg of the skinned muscle fibres were exposed to heme (300 µM), DTT (50 mM) or DTDP (2.5 mM) as indicated in (**A**). Then cysteinyl SH groups were labelled by biotin and the labelled proteins were analyzed by SDS-PAGE. Biotinylated proteins were visualized by streptavidin and shown in (**A**). The apparent positions of molecular weight standards are shown on the left. The position of some proteins was tested on the same membranes by antibodies specific for myosin heavy chain (MHC), myomesin-2, myosin-binding protein C (MyBPC) or α-actinin. Results of typical western immunoblots are shown on the right side of (**A**). Results of the densitometric analyses of the bands are given in (**B**–**E**). The apparent molecular weights and the identity of the co-migrating proteins are indicated in the headers of the graphs. Treatments are the same as described in (**A**). Data are from three independent assays and were normalized to the control. Bars show means ± S.E.M. and statistical significance is shown by asterisks (*) when *p* < 0.05.

Next, the molecular mechanisms of heme-induced functional alterations were further investigated. To this end, the oxidative effects of heme were tested in skinned skeletal muscle fibres. Protein cysteinyl SH-group content was determined by a SH-specific biotinylation assay (Figure 2) before and after heme exposures (300 µM). Heme evoked an apparent decrease in cysteinyl SH contents at the level of several proteins that was reversed to a large degree by dithiothreitol (DTT) (50 mM, Figure 2A, lane 2); thus, consistent with oxidation of protein SH-groups (Figure 2A, lane 3). The above findings were reminiscent of those observed following incubations in the presence of the SH-specific oxidative agent 2.5 mM 2,2′-dithiodipyridine (DTDP) in the absence and presence of DTT (Figure 2A, lanes 4 and 5). Protein bands affected by heme treatments were identified by conventional western immunoblot (Figure 2A, right) and mass spectrometry (Table 1). Heme evoked a partial oxidation of proteins co-migrating with myosin heavy chain (MHC from 100% to 55.1 ± 7.9%; in relative units), myomesin-2 (from 100% to 30.9 ± 13%), myosin-binding protein C (MyBPC from 100% to 51.2 ± 11%) and α-actinin (from 100 to 46.9 ± 5.2%) (Figure 2B–E). However, the level of oxidation was generally lower than that for the specific SH-oxidant DTDP; furthermore, heme-induced SH oxidation was not fully reversed by DTT.

**Figure 3:**
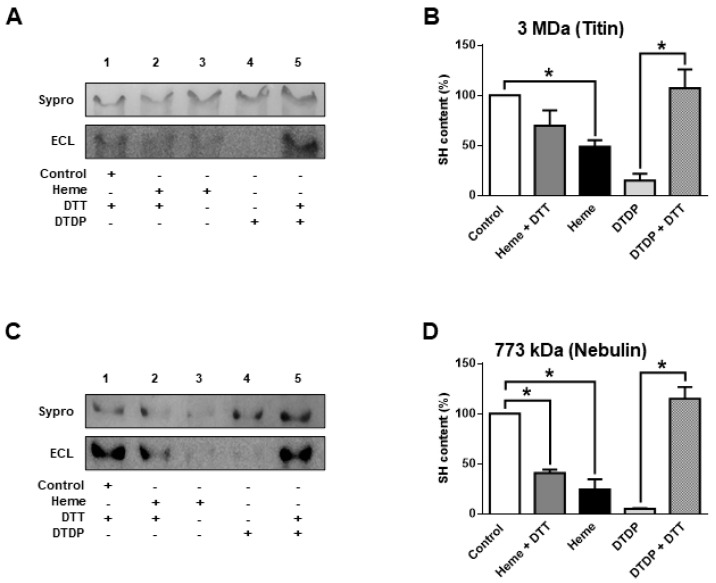
Heme-induced SH group oxidation in large myofibrillar proteins. Human skeletal muscle fibres were treated as described in Figure 2. Proteins were solubilized and subjected to agarose strengthened SDS-polyacrylamide gels. Proteins were stained by Sypro-Ruby (total protein), and biotinylation was tested by streptavidin (**A**,**C**). Representative gel pictures (**A**,**C**) and values from the densitometric analyses are shown on bar graphs (**B**,**D**). The apparent molecular weights of the bands and the identified proteins co-migrating with these bands are indicated in the headers. Data represent the results of three independent experiments and were normalized to the control. Bars show means ± S.E.M. and statistical significance is shown by asterisks (*) when *p* < 0.05.

The giant sarcomeric titin is a molecular determinant of F_passive_. Hence, titin oxidation was also tested in separate biochemical assays (Figure 3A,B).

Heme (300 µM) evoked a partial titin oxidation similarly to other sarcomeric proteins: i.e., partial oxidation (when compared to that evoked by DPDT) with limited reversibility by DTT. A similar characteristic was observed for the protein co-migrating with nebulin (Figure 3C,D).

**Figure 4:**
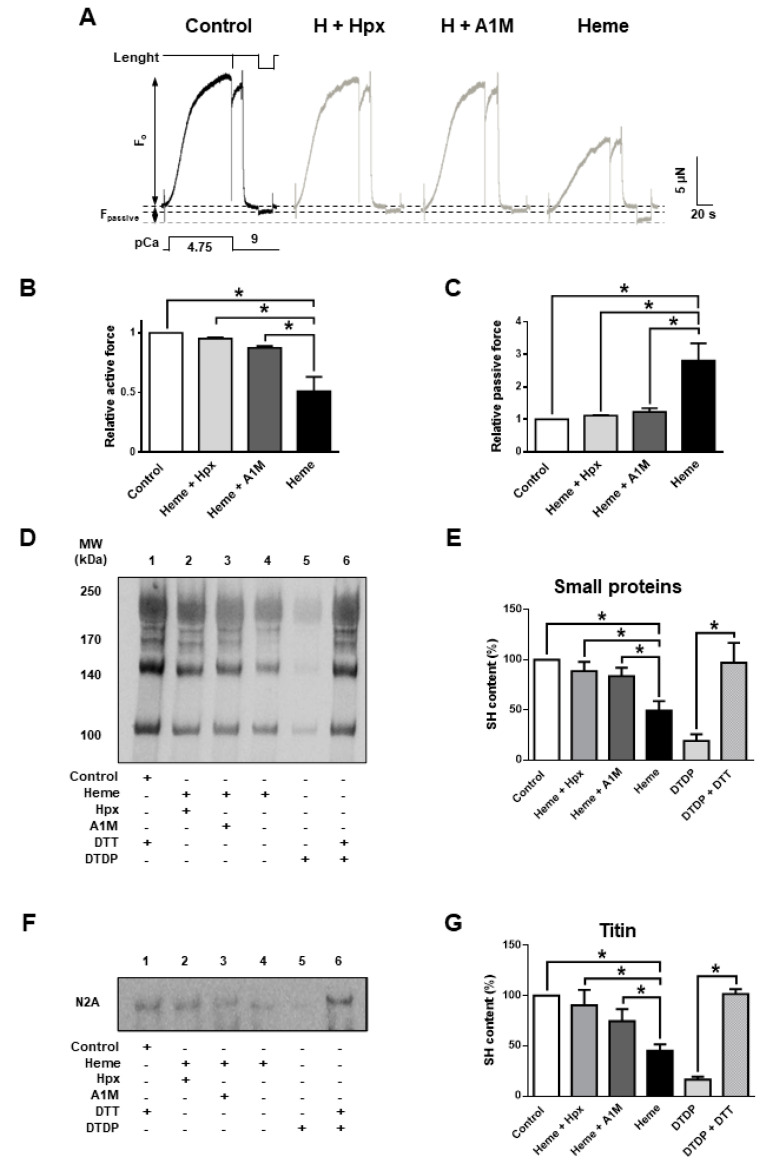
Prevention of heme evoked deterioration by heme binding proteins or heme “scavengers”. Human skeletal muscle fibres were treated with heme as described in Figure 1. In addition, heme-protein complexes were pre-treated with heme-hemopexin (Hpx) and heme-α-1-microglobulin (A1M) complexes as shown. (**A**) shows original force recordings. Active (**B**) and passive (**C**) force values are normalized to the control and are shown in bar graphs. Biotinylation was performed as described in Figure 2. Representative blots are shown in (**D**,**F**), while the results of the densitometric analyses are shown in bar graphs (**E**,**G**). Data represent the results of three independents experiments and were normalized to the control. Bars show means ± S.E.M. and statistical significance is shown by asterisks (*) when *p* < 0.05.

Heme scavenger proteins are protective against free heme in vivo in many biological fliuds including plasma. Thus, their presence is expected to prevent heme-mediated deterioration of contractile function. In hemolytic conditions if these proteins are depleted then supplementation could be therapeutic. Both Hpx (20 µM) and A1M (10 µM) and prevented the heme (20 µM) evoked decrease in F_active_ and the heme evoked increase in F_passive_ (Figure 4A–C) in vitro.

Heme treatment alone decreased F_active_ to 51.2 ± 6% and evoked a 2.8 ± 0.3-fold increase in F_passive_ (whereas heme:Hpx is 1:1 and heme:A1M is 1:2). The protective effects of A1M and Hpx were paralleled by reduced levels of cysteinyl SH oxidation of sarcomeric proteins, as both A1M and Hpx inhibited heme evoked SH oxidation in small sarcomeric proteins (Figure 4D,E) and also in titin (Figure 4F,G).

**Figure 5:**
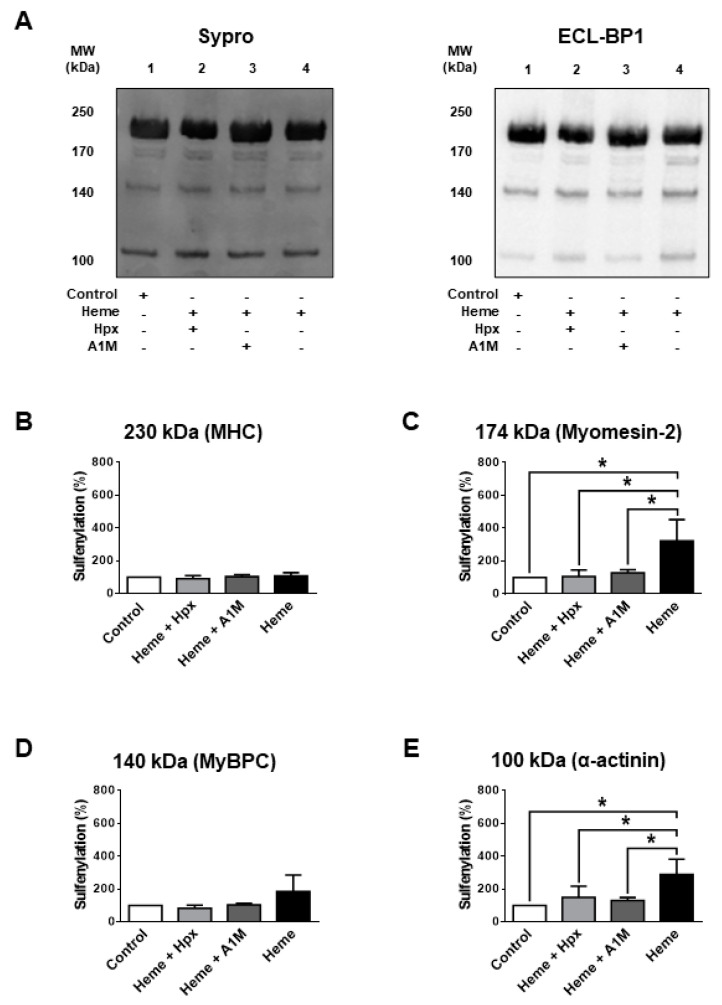
Sulfenylation of skeletal muscle proteins with low molecular weights by heme. Human skeletal muscle fibres were isolated and treated as described in Figure 2. Proteins were subjected to SDS-PAGE and plotted onto nitrocellulose membranes. Proteins were visualized by the non-specific Sypro Ruby dye and the same membranes were subsequently processed with sulfenyl specific labelling. (**A**) shows representative membranes. Bar graphs (**B**–**E**) show the results of densitometric analyses. The headers of the graphs indicate the molecular weights of the evaluated bands and the identified proteins. Data represent three independents experiments and were normalized to the control. Bars show means ± S.E.M. and statistical significance is shown by asterisks (*) when *p* < 0.05.

**Figure 6:**
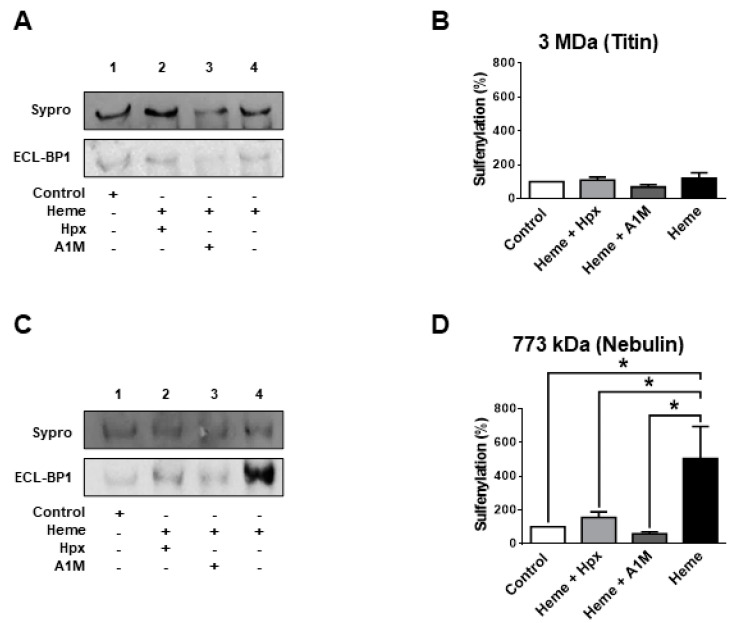
Sulfenylation of skeletal muscle proteins with high molecular weights by heme. Human skeletal muscle fibres were isolated as described in Figure 2. Proteins were subjected to agarose strengthened SDS-PAGE and transferred to nitrocellulose membranes. Membranes were stained for protein by Sypro Ruby and sulfenylation was detected by western immunoblot (labelled by ECL-BP1). Representative membranes are shown in panel (**A**,**C**). Results of densitometric evaluations are shown in panel (**B**,**D**). Data illustrate the results of three independents experiments and were normalized to the control. Bars show means ± S.E.M. and statistical significance is shown by asterisks (*) when *p* < 0.05.

We next addressed why it was intriguing that DTT only partially reversed heme-evoked protein SH modification. This finding implied that heme exposures caused protein alterations that involved not only the reversible protein SH oxidations (such as in the case of DTDP) but additional ones that limited the availability of cysteinyl SH groups for the biotinylation agent. One of these potential mechanisms is protein sulfenylation (SOH formation). SOH formation was tested for sarcomeric proteins with relatively low (Figure 5) and large molecular weights (Figure 6) in heme treated (300 µM) human skinned skeletal muscle fibres. Heme was without effects on SOH levels in MHC (Figure 5B), MyBPC (Figure 5D) and titin (Figure 6B), while it increased SOH levels in myomesin-2 (3.2 ± 0.7-fold increase, Figure 5A,C), in α-actinin (2.9 ± 0.5-fold increase, Figure 5A,E) and in nebulin (5.0 ± 1.0-fold increase, Figure 6C,D).

## 3. Discussion

Here, we describe our investigations on the mechanisms of heme-induced toxic effects in human skeletal muscle preparations. Our research has identified several myofilament proteins as potential molecular targets during pathologies of both cardiac and skeletal muscles due to increased free heme concentrations. Notably, the applied heme concentrations were comparable to those found in pathological conditions, e.g., SCD (20 µM) [16] or thalassemia (50–280 µM) [21] or employed by others under test conditions (1.5–300 µM) [18].

Currently, there is only limited information available on the effects of free heme on skeletal muscle function. Avissar et al. published the first evidence on intermolecular interactions between heme and actin in rabbit skeletal muscles [18]. These authors found that at relatively high heme concentrations (300 μM) in vitro G-actin polymerization can be disturbed. Lund et al. investigated the oxidation of myosin by hemoproteins (e.g., horseradish peroxidase and meta-myoglobin) in porcine longissimus dorsi muscle [17]; and they proposed that hemoproteins (e.g., myoglobin) generate disulfide bonds and promote di-tyrosine formations on myosin.

Results of our recent study in isolated cardiomyocytes implicated that heme-induced oxidation may limit the physiological function (i.e., contractility) in parallel with oxidations of α-actinin, filamin C and titin. In this study the concentration dependencies of free heme on mechanical and biochemical properties of muscles and their individual proteins were studied in detail. Similar to our present observations, the mechanical alterations developed at low heme concentrations. Nevertheless, due to a relatively low signal to noise ratio of the parallel biochemical assays, the recognition of significant heme-induced protein alterations required relatively high heme concentrations [19]. Our present findings in skeletal muscle fibres confirm and extend the above observations, whereby heme-evoked that was partially reversible cysteinyl SH oxidations of several muscle proteins was prevented by heme-binding proteins. Importantly, here we report the irreversible oxidation (by sulfenylation) of three myofibrillar proteins. First, α-actinin is a structural protein, which can be oxidized leading to diminished F_active_ and increased F_passive_ in cardiomyocytes [22]. Second, nebulin stabilizes F-actin and regulates muscle contraction at the level of crossbridges [23]. Third, meromyosin-2 shares the same structural function of α-actinin providing stability during muscle contraction by its interaction with MHC and it functions as a spring (similar to titin) [24]. Thus, collectively, our results convincingly illustrate the involvement of the above sarcomeric proteins in heme-induced toxic alterations. Unfortunately, to what extent these oxidative protein modifications limit sarcomeric function individually remains to be fully determined. For example, DTDP-evoked oxidation completely abolished F_active_ together with a pronounced α-actinin oxidation in cardiomyocytes. However, this had only a limited effect on F_passive_ in cardiomyocytes (1.2-fold increase) [22]. Therefore, we propose that the heme-induced mechanical alterations are mediated by the interaction of several target proteins in human skeletal muscle fibres.

Interestingly, the effects of heme on F_passive_ appeared to be different in cardiac and skeletal muscles. In particular, heme evoked a five-fold increase in F_passive_ in cardiomyocytes [19], and about a 3-fold increase in skeletal muscle fibres under comparable experimental conditions. Differences in the composition of the titin isoforms between cardiac and skeletal muscles may potentially explain this difference: skeletal muscles express the N2A titin isoform that has longer elastic segments than the N2B cardiac titin isoform [25]. Hence structural differences between titin isoforms may modulate the extent of their heme induced oxidations.

Here, we report that heme-evoked oxidation of myofibrillar proteins results in a decreased force production together with an increased “stiffness” in association with oxidative modifications of myofibrillar proteins. It remains to be fully clarified how far the effects observed under our in vitro settings can be extrapolated to human cardiac and skeletal myopathies. Furthermore, one of the conditions, in which heme is released, is SCD, characterized by muscle weakness commonly explained by anemia [26]. Children suffering from SCD typically present with low protein stores and muscle wasting [27] in association with reduced capacities for exercise and lower strength. Nevertheless, the underlying mechanisms are only partially understood [28]. Interestingly, the SCD-related decrease in muscle force develops without tissue necrosis, inflammatory stress, or fibrosis [29]. Moreover, the estimated free heme level can reach 20 µM in SCD [16]. This heme concentration evoked a decrease in Ca^2+^ dependent force production of about 50% here. Thus, we suggest that in SCD the heme contributes to the decreased muscle strength.

Another myopathy with elevated heme is present in patients with chronic heart failure is skeletal muscle myopathy, and this is considered as a main cause of exercise intolerance in these patients [30]. However, the molecular pathophysiology of skeletal muscle myopathy is not well understood. This pathology is characterized by multiple alterations, like mitochondrial dysfunction, systemic inflammation, oxidative stress, and decreased muscle strength (isometric force), and atrophy of oxidative fibres [31]. It was shown that heart failure also induces skeletal muscle myopathy, and free heme was detected in patients with chronic heart failure [32], thus implicating a potential cause for the mechanical impairment of the myocardium [19]. Taken the above findings and the results of this study together we speculate that free heme causes skeletal muscle contractile dysfunction by oxidation of myofibrillar proteins, mitochondrial dysfunction, and oxidative stress [11,12,13,19].

Hemolytic diseases, such as observed e.g., in thalassemia [21] can also associate with the release of free heme, thereby promoting pro-oxidant conditions [33,34]. Accordingly, Hpx has been shown to confer a protective effect by removing free heme from the circulation during hemolytic conditions [35]. In this study, equimolar concentrations (20 μM) of heme and Hpx were employed, and we found that the heme-induced mechanical changes were largely prevented by these two proteins. Our observations are largely comparable to that reported by Grinberg et al. where the catalytic reactivity of heme was probed in the presence of Hpx [36]. Collectively, these data implicate that Hpx can effectively scavenge heme during in vivo conditions. Nevertheless, during a number of pathologic conditions (e.g., acute intermittent porphyria, hemolytic anemia, SCD and ischemia-reperfusion injury) the amount of released heme can exceed the binding capacity of Hpx [37]. Accordingly, fulminate rhabdomyolysis [15] and severe sepsis can deplete Hpx and give rise to heme-mediated pathologies and oxidation of skeletal muscle proteins as reported here. Moreover, in some neuromuscular diseases such as polymyositis/dermatomyositis and myasthenia gravis increased levels of circulating Hpx have been associated with myoglobin release [14]. Recently A1M has been also identified as a molecule with a potential for scavenging heme [38], free radicals [39], moreover as a reducing agent of oxidized molecules [40,41]. Here we confirmed that A1M has similar protective effects against heme toxicity as Hpx. Taken together, these data on heme evoked deteriorations of skeletal muscle function provide additional clinical situations where these two extracellular plasma heme binding proteins limit heme toxicity, However, it is to be stressed that although heme-binding is probably an important element of the A1M effect, the protective mechanism of A1M in hemolytic and muscular diseases has not yet been fully defined.

In conclusion, heme induces protein SH oxidation in human skeletal muscle fibres, which results in a reduction in Ca^2+^-regulated contractile force in association with a pronounced increase in muscle rigidity. Heme-mediated contractile effects were prevented by the heme binding proteins, Hpx and A1M, further supporting that these proteins can be used to limit the deleterious effects of free heme in myopathies. Finally, our data suggest that heme release may be directly responsible for skeletal muscle weakening (often observed and related to anemia), which can be prevented by heme scavengers.

## 4. Materials and Methods

### 4.1. Ethical Approval

The experiments performed in this study complied with the Helsinki Declaration of the World Medical Association and were approved by the Institutional Ethical Committee at the University of Debrecen, Hungary ((ETT TUKEB 21752-6/2014/EKU (201/2014)) and by the Hungarian Ministry of Health (HBR/052/00766-2/2014).

### 4.2. Skeletal Muscle Tissue Samples

Small human skeletal muscle samples (gluteus medius) were obtained during hip replacements due to technical reasons from three male patients without muscle diseases. After excision, samples were placed in plastic tubes, frozen in liquid nitrogen and then stored in deep freezer at −70 °C. Samples were defrosted and shredded with a high-speed homogenizer in isolating solution containing: 100 mM KCl, 1 mM MgCl_2_, 2 mM EGTA, 10 mM imidazole and 4 mM ATP, pH 7.0 supplemented with 0.5 mM phenylmethylsulfonyl fluoride (PMSF, Sigma, St. Louis, MO, USA). Skeletal muscle fibres were chemically permeabilized (skinned) in isolating solution also containing 0.5% triton X-100 (Sigma, St. Louis, MO, USA) for five minutes on ice. Finally, preparations were washed three times in isolating solution, centrifuged at 0.4× rcf for one minute and kept at 4 °C.

### 4.3. Force Measurements in Single Skeletal Muscle Fibre Preparations

Permeabilized segments of a single skeletal muscle fibres were fixed with silicone adhesive (DAP Aquarium, Baltimore, MD, USA) between two thin stainless-steel needles. One needle was attached to an electromagnetic motor (Aurora Scientific Inc., Aurora, ON, Canada) and the other to a force transducer (SensoNor, Horten, Norway). Sarcomere length was measured in isolating solution at 15 °C and set to 2.3 µm [42]. The composition of test solutions (activating and relaxing) was calculated as described previously [43,44]. Relaxing test solution had the following composition: 7 mM EGTA, 6.41 mM MgCl_2_, 10 mM NN-bis (2-hydroxyethyl)-2-aminoethanesulfonic acid (BES) and 37.11 mM KCl, 6.94 mM MgATP, 15 mM creatine phosphate, pH 7.2; protease inhibitors: 10 μM E-64, 40 μM leupeptin and 0.5 mM PMSF. Activating solution had the same compositions as relaxing solution except for 7 mM CaEGTA replacing 7 mM EGTA. The ionic equivalent was adjusted to 150 mM with KCl (ionic strength of 186 mM). The pCa (−log [Ca^2+^]) values for the activating and relaxing solutions were 4.75 and 9, respectively. Intermediate free [Ca^2+^] levels solutions were obtained by mixing relaxing and activating solutions.

Isometric force production was followed at various Ca^2+^ concentrations. When a constant force level was attained, segment length was reduced by 20% within two milliseconds and then the preparations were quickly restretched. As a result, the force first dropped from the peak isometric level to zero (difference = total peak isometric force, F_total_) and then started to redevelop. About five seconds after the onset of force redevelopment, the preparation was returned to the relaxing solution, where a shortening to 80% of the original length with a long slack duration (eight seconds) was performed to determine passive (Ca^2+^ independent) force (F_passive_). Active (Ca^2+^ evoked) force (F_active_) was calculated by subtracting F_passive_ from F_total_ for each Ca^2+^ concentration. Force was normalized for the cross-sectional area, calculated from the width and height of permeabilized muscle segments.

The mechanical measurements started with a first activation at maximal calcium concentration (pCa 4.75), then sarcomere length was readjusted to 2.3 µm, if necessary. Second activation at pCa 4.75 was used to calculate F_total_. Muscle preparations were incubated in the presence of various in vitro test conditions in a small droplet (100 µL) of solution on the microscope stage for 20 min under relaxing conditions at room temperature. Mechanical forces were recorded before and after test incubations. F_active_ at submaximal levels of activation was normalized to that at maximal activation so as to characterize the pCa_50_.

### 4.4. Heme Treatment

A 4 mM stock solution of hemin chloride (pH 12.65, 15 °C Sigma-Aldrich St. Louis, MS, USA) was freshly prepared each day in a dark room and diluted in relaxing solution (to reach a final concentration between 20 μM and 300 μM). Hemopexin and human recombinant A1M (prepared as described previously [45,46]) were dissolved in relaxing solution in a molar ratio (protein:heme) of 1:1 and 1:2, respectively.

### 4.5. Identification of Protein SH Groups in Myofilament Proteins

Permeabilized human skeletal muscle fibre segments (about 25 mg) were incubated for 20 min in the presence of test solutions under the same conditions employed for force measurements. Thereafter, test solutions were removed by three washing steps. The pellet fraction was exposed to EZ-Link Iodoacetyl-LC-Biotin (Thermo Scientific, Rockford, IL, USA) at room temperature for 60 min in a darkened room in a reaction buffer (50 mM Tris-HCl and 5 mM EDTA, pH 8.3) to biotinylate the free (i.e., reduced) SH groups of proteins. Biotin was dissolved in dimethylformamide (DMF, Sigma-Aldrich, St. Louis, MO, USA) and homogenized by vortex for 30 min at room temperature. The reaction was stopped, and the preparations were solubilized by the addition of urea buffer (8 M urea, 10% glycerol, 2 M thiourea, 75 mM DTT, 3% SDS, 50 mM Tris-HCl, 10 µM E64, 40 µM leupeptin and 0.5% bromo-phenol blue at pH 6.8) for at least 45 min. Total protein content in solubilized samples was determined by a dot blot-based method and adjusted to 1 mg/mL. Subsequently, lanes on 2.1% and 4% polyacrylamide gels were loaded with similar amounts of solubilized protein (10 μg). Proteins were transferred to nitrocellulose membranes and the protein amounts were determined by fluorescent Sypro Ruby Protein Blot Stain (Invitrogen, Eugene, OR, USA). Free protein binding sites on the membranes were blocked by milk powder (10%) for one hour and then streptavidin-peroxidase conjugate (Jackson ImmunoResearch, West Grove, PA, USA) was used (60 min incubation) to identify the biotinylated proteins. Enhanced chemiluminescence (ECL, Bio-Rad Laboratories, Inc. Hercules, CA, USA) was applied to visualize the signal intensities of biotin labeled SH groups. Finally, the intensities were normalized for those stained by Sypro Ruby Protein Blot Stain in the same location. Exposures of human permeabilized skeletal muscle fibres to the SH-oxidant 2,2’-dithiodipyridine (DTDP; Sigma-Aldrich, St. Louis, MS, USA) served as internal controls.

### 4.6. LC-MS/MS Based Protein Identification

Bands in Coomassie blue stained gels were dissected. Gel pieces were incubated with 20 mM dithiotreitol (DTT) for 60 min. This was followed by alkylation with a solution containing 55 mM iodoacetamide in the dark for 45 min. Then overnight trypsinization was performed using TPCK treated stabilized MS grade bovine trypsin (ABSciex, Farmingham, MA, USA) at room temperature and the digested peptides were extracted and lyophilized. The peptides were re-dissolved in 10 µL 1% formic acid and used for LC-MS/MS analysis. Before mass spectrometry analysis peptides were separated using a 90 min water/acetonitrile gradient and 300 nL/min flow rate on an EasynLC (Bruker, Bremen, Germany) nano HPLC. The separation was done on a 10 cm reverse phase Easy-Column (ID 75 µm, 3 µm, 120 Å, ReproSil-Pur C18-AQ, Thermo Scientific, Waltham, MA USA). Mass spectrometry analysis was performed on a 4000 QTRAP (ABSciex, Farmingham, MA, USA) mass spectrometer, operating in positive ion mode (spray voltage: 2800 V, ion source gas: 50 psi, curtain gas: 20 psi, source temperature: 70 °C). Information Dependent Acquisition was applied; first, a survey scan (+EMS: 440-1400 amu) was recorded, followed by an enhanced resolution scan (ER) to determine the charge state of the two most intensive ions. Using this information, the proper collision energies were calculated by the Analyst v. 1.4.2 software (ABSciex, Farmingham, MA, USA); the selected parent ions were fragmented by collision induced dissociation (CID) and the product ions were scanned (EPI MS/MS). The acquired LC-MS/MS data were used for protein identification using the ProteinPilot 4.5 software (ABSciex Farmingham, MA, USA) applying Paragon algorithm and the UniProtKB/Swiss-Prot database (v. 2014. 06. 11, 545536 entry). Peptide sequences having at least 95% confidence were accepted and at least two peptides were used for protein identification.

### 4.7. Sulfenic Acid Labeling

Permeabilized muscle fibres were treated similarly to those described for the identification of protein SH groups. Briefly, preparations were exposed to 2 μM biotin-1-3-cyclopentanedione (BP1) (Kerafast, Boston, MA, USA) in 50 mM Bis-Tris citric acid buffer (pH 5.5) [47]. After 90 min of labeling with BP1, muscle fibres were solubilized in urea buffer for 45 min. Then 10 µg protein homogenates were loaded on 2.1 and 4% polyacrylamide gels and after SDS-PAGE the separated proteins were transferred onto nitrocellulose membrane. Sypro Ruby Protein Blot Stain (Thermo Fisher Scientific Inc., Waltham, MA, USA) were used to determine the protein amounts. Free protein binding sites on the membranes were blocked by milk powder (10%) for one hour and then streptavidin-peroxidase conjugate (Jackson ImmunoResearch, West Grove, PA, USA) was used (60 min incubation) to identify the biotinylated proteins. SOH groups were identified by ECL method and normalized for those assessed with Sypro Rubi Protein Blot Stain.

### 4.8. SDS-PAGE and Western Immunoblot

Proteins of muscle homogenates were separated on 2.1% SDS-polyacrylamide gels or on 4% SDS-polyacrylamide gels strengthened with agarose (0.5%) and 10 µg protein homogenates were loaded in each lane. Separated proteins were blotted onto nitrocellulose membranes and blocked for 60 min with 2% bovine serum albumin in Tris-buffered saline (20 mM Tris, 0.1% Tween, pH 7.5). The membranes were then incubated with antibodies against nebulin (mouse monoclonal 1 µg/mL Abcam PLC. Cambridge, UK), MHC (mouse monoclonal 1:10,000, Sigma Aldrich, St. Louis, MS, USA), myomesin-2 (rabbit polyclonal 1 µg/mL, Abcam PLC. Cambridge, UK), MYBPC (rabbit polyclonal 1 µg/mL, Abcam PLC. Cambridge, UK) and α-actinin (mouse monoclonal 1:100,000, Sigma Aldrich, St. Louis, MS, USA). Membranes were washed and subsequently incubated for 60 min at room temperature in the presence of a peroxidase-labeled anti-mouse specific secondary antibody (Sigma Aldrich, St. Louis, MS, USA 1:40,000) for all proteins except for myomesin-2 in which case a goat anti-rabbit (Sigma Aldrich, St. Louis, MS, USA 1:300) was used. Signal intensities were visualized by the ECL method in DNR Bio-imaging Systems (MF-ChemiBis 3.2, Jerusalen, Israel).

### 4.9. Data Analysis

Peak active force levels measured during repeated activation/relaxation cycles at various Ca^2+^ concentrations were fitted to a modified Hill equation (see Equation (1)):

(1) $$F_{total}=F_{passive}+\frac{F_{max}{\left[Ca^{2+}\right]}^{nHill}}{\left(pCa_{50}^{nHill}+{\left[Ca^{2+}\right]}^{nHill}\right)}$$

where *F_max_* is the maximal force, *F_passive_* is the passive force, *F_total_* = *F_max_* + *F_passive_*, [*Ca*^2+^] is the calculated *Ca*^2+^ concentration, *nHill* is a constant, and *pCa*_50_ corresponds to the [*Ca*^2+^] at which *F_total_* − *F_passive_* = *F_max_*/2.

Values are mean ± S.E.M. Mean values of force measurements reflect the results of at least 5 independent observations from 3 different human muscle samples. Statistical significance was determined using one-way ANOVA followed by Tukey multiple comparison tests. Statistically significant differences were considered when *p* < 0.05. GraphPad Prism^®^ (v. 6.01) (GraphPad Software Inc., San Diego, CA, USA) was used to plot and analyze the data.

## Figures and Tables

**Table 1:** Identification of biotinylated proteins by mass spectrometry. Biotinylated bands were excised from the gels and digested by trypsin. The peptides were evaluated by mass spectroscopy and the identified peptide sequences and the original proteins from which they were extracted are shown in the table. The Protein Data Bank identifiers are also shown for the identified proteins.

Protein	Peptide Sequence
Titin Q8WZ42	AGDTIVLNAISILGKPLPK
	VGEAFALTGR
	GLLQAFELLK
	STDFDTFLR
	IVPGVIGLMR
Nebulin P20929	SDAIPIVAAK
	IYETTTTR
	VVLDTPEYR
Myosin 7 P12883	DLEEATLQHEATAAALR
	LASADIETYLLEK
	NLTEEMAGLDEIIAK
	NNLLQAELEELR
	SVNDLTSQR
	VGNEYVTK
	LLSTLFANYAGADAPIEK
	VIQYFAVIAAIGDR
	MFNWMVTR
	DLEEATLQHEATAAALR
Myomesin-2 P54296	LLCETEGR
	LTVELADHDAEVK
	NGLDLGEDAR
	VGQHLQLHDSYDR
	VIDVPDAPAAPK
	YGLATEGTR
	ATNLQGEAR
	PEPGKKPVSAFSK
	RVHSGTYQVTVR
	QGVLTLEIR
	YIFESIGAK
MyBPC Q00872	AVNAAGASEPK
	NSETDTIIFIR
	AVNAAGASEPK
	VGEDITFIAK
α-actinin P35609	VLAVNQENER
	ATLPEADGER
	ILASDKPYILAEELR
	VGWELLLTTIAR
	VGWELLLTTIAR

## Abbreviations

[*Ca*^2+^]	Calcium concentration
A1M	α1-microglobulin
DMF	Dimethylformamide
DTDP	2,2′-dithiodipyridine
DTT	Dithiothreitol
ECL	Enhanced chemiluminescence
EGTA	Ethylene glycol tetra-acetic acid
*F_active_*	Active force
*F_passive_*	Passive force
*F_total_*	Total peak isometric force
HO-1	Heme oxygenase 1
Hpx	Hemopexin
IC_50_	Half maximal inhibitory concentration
MHC	Myosin heavy chain
MyBPC	Myosin-binding protein C
*pCa*	Log [*Ca*^2+^]
*pCa*_50_	*Ca*^2+^ sensitivity of isometric force production
PMSF	Phenylmethylsulfonyl fluoride
ROS	Reactive oxygen species
S.E.M.	Standard error of mean.
SCD	Sickle cell disease
SH	Sulfhydryl groups
SOH	Sulfenic acid
